# A Conceptual Model for Ambient AI Adoption: Perspectives From Academia and Industry

**DOI:** 10.2196/91098

**Published:** 2026-08-28

**Authors:** Joshua Biro, Jesse M Pines, Sudha Jayaraman, Vamsi Chagari, Raj Ratwani

**Affiliations:** 1 National Center for Human Factors in Healthcare, Medstar Health Research Institute, 3007 Tilden St. NW Suite 6N, Washington DC, United States, 1 301-542-3073; 2 Department of Family Medicine, Georgetown University School of Medicine, Washington DC, United States; 3 US Acute Care Solutions, Arlington, VA, United States; 4 Suki, Redwood City, CA, United States; 5 Department of Emergency Medicine, Georgetown University School of Medicine, Washington DC, United States

**Keywords:** ambient artificial intelligence, ambient AI, artificial intelligence, AI, adoption, implementation, evaluation

## Abstract

Ambient AI technologies are increasingly marketed as solutions to reduce clinician burden and improve care efficiency; however, real-world performance varies widely across clinical settings. Health care provider organizations face challenges in determining which aspects of ambient AI performance matter most and how to obtain meaningful information about those aspects from vendors or through internal evaluation. This article presents a shared mental model to guide health system leaders in conceptualizing ambient AI performance across 3 interdependent dimensions: technical, interface, and system level. For each dimension, we outline the types of information relevant to assessment; what vendors should reasonably be expected to provide; and how health care provider organizations can conduct their own evaluations to contextualize, verify, or supplement vendor claims. By integrating both vendor and health system perspectives, this work offers a grounded, practical structure to support organizations of all sizes in understanding and making informed decisions about ambient AI technologies.

## Introduction

Ambient AI technologies for documentation in health care have shown the potential to reduce documentation burden, enhance workflow efficiency, improve patient experience, and increase patient safety [1-5]. This potential has driven rapid adoption, with health care provider organizations now facing consequential decisions about whether to adopt these technologies, which products to select, and how to integrate them into existing clinical workflows.

In making these decisions, health care provider organizations must rely on limited and difficult-to-interpret information. Early evaluations show mixed results in achieving anticipated benefits, with little explanation for why outcomes are not being consistently realized [6,7]. While vendor claims, internal evaluations, and reports from early adopters provide signals about performance, these signals are often inconsistent, incomplete, or not directly comparable across organizations. As a result, health care provider organizations must make consequential decisions regarding ambient AI despite substantial uncertainty.

An important driver of this uncertainty is the underlying complexity of ambient AI performance. Performance is shaped by multiple interdependent factors spanning the underlying model, the interface that mediates clinician interaction, and the organizational context in which the tool is deployed. However, existing assessments of ambient AI technologies often determine these dimensions in isolation—focusing on evaluating model accuracy or determining workflow integration—without accounting for how they jointly shape real-world performance [8,9]. This fragmentation makes it difficult to interpret observed outcomes, attribute sources of variation, or compare technologies in a meaningful way. For example, an inaccurate clinical note might reflect limitations in model training data, usability issues that hinder effective clinician review, or environmental factors such as poor audio capture. Without a conceptual structure that distinguishes these sources of variation, health care provider organizations may struggle to select the right technology, interpret performance information, or design effective monitoring processes.

## Shared Mental Model for Ambient AI Performance Assessment

### Overview

**Figure 1.**
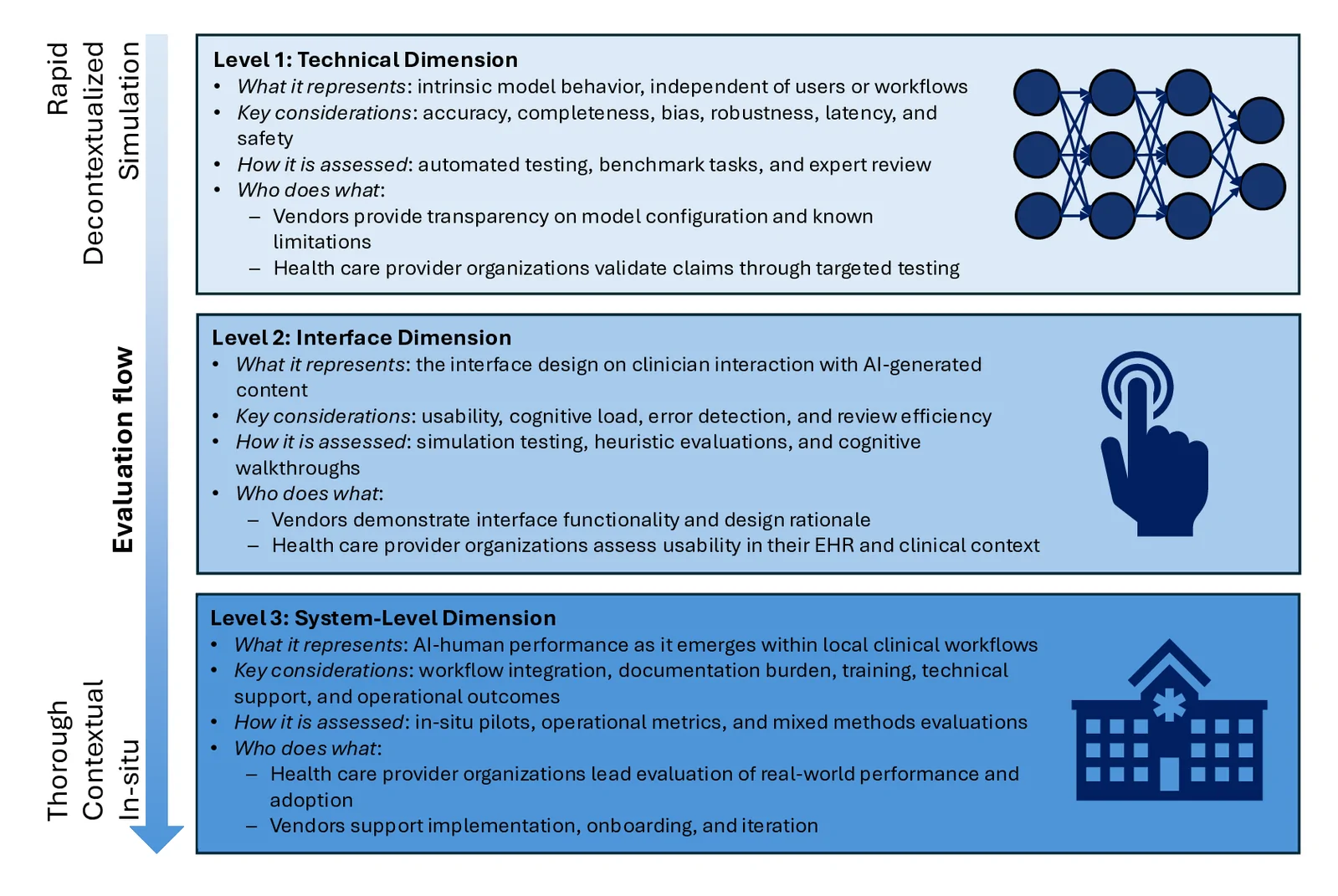
Tri-level model for conceptualizing ambient AI performance. EHR: electronic health record.

To address these challenges, we propose a shared mental model that conceptualizes ambient AI performance into 3 interdependent dimensions: technical, interface, and system level (Figure 1). This framework provides a structured way to understand how different factors contribute to observed performance, enabling health care provider organizations to more effectively interpret performance signals, assess vendor claims, and compare technologies. Rather than prescribing a single protocol, this model clarifies what aspects of performance can be assessed at each level, what information vendors can reasonably be expected to provide, and how to perform internal assessments to validate vendor claims or contextualize the technology within an organization’s system. By incorporating perspectives from both health system implementers and industry, this model provides a practical and coherent structure to support organizations of all sizes in understanding and making informed decisions about implementing ambient AI documentation tools.

### Level 1: Technical Dimension

The technical dimension reflects the fundamental capabilities and limitations of the underlying automatic speech recognition (ASR) and AI models that generate transcripts and draft clinical notes. These capabilities determine the model’s intrinsic capacity for accurate, complete, and clinically appropriate output prior to any interaction with users or clinical workflows.

This combination of capabilities also varies across vendors who might own all or some parts of these technologies or none at all. Health system awareness of these technical elements is critical as they represent key underlying sources of variation in performance across products and settings. Understanding these characteristics provides valuable context when comparing different technologies and interpreting their limitations, and this awareness also helps organizations distinguish technical constraints from issues that arise at the interface or system levels, set realistic expectations for performance, and identify where additional evaluation or monitoring is required. Importantly, clarity about the capabilities and limitations of the underlying ASR and AI models can also guide implementation efforts by helping clinicians and staff understand what the technology can reliably do and where additional review is essential.

Assessing this dimension requires understanding the specific components that shape model behavior. These include the underlying foundation large language models (LLMs) and version in use (eg, Open AI’s GPT-5.5 vs GPT-4o or Google’s Gemini 3.1 Pro vs Gemini 3 Flash), the prompting or orchestration strategies applied (eg, zero-shot, few-shot, chain-of-thought, in-context learning, and retrieval-augmented generation), and the characteristics of the data used for training (eg, general-purpose, domain-specific, specialty-specific, and region-specific datasets). Vendors may use any, all, or some combination of these aspects to create their outputs and typically study the effectiveness of different combinations in internal testing prior to releasing new versions of their products. Training data characteristics, including the clinical contexts, populations, and demographics represented, are particularly important for assessing how well a model is likely to generalize to a given patient population. Vendors typically train models on broad, heterogeneous datasets that may not adequately represent the demographic, geographic, and specialty-specific characteristics of particular health care provider organizations. However, training data can often overrepresent certain populations and regions while underrepresenting others, and disclosure of dataset composition remains inconsistent across the industry. Consequently, a model’s strong performance on general benchmarks may not reliably predict its effectiveness within a specific clinical context.

Key performance metrics in the technical dimension can include word error rate, accuracy, bias, reliability, efficiency, hallucination rate, omission rate, safety (detection of potentially harmful outputs), latency, completeness, relevance, and robustness (consistency across similar inputs and graceful handling of edge cases). Prior technical evaluations provide useful examples of how some of these metrics, such as accuracy, omission, error rates, potential harm, and note quality, can be defined, measured, and reported in practice [10,11]. These metrics are not independent and often reflect different underlying components of the system. For example, transcription inaccuracies may arise from limitations in ASR performance, particularly in the presence of diverse accents, background noise, or multispeaker environments. In contrast, hallucinations or unsupported clinical statements are more commonly associated with LLM behavior, including model capability, prompting strategies, and the scope and representativeness of training data. Understanding these relationships can help health care provider organizations interpret observed errors and identify which aspects of a system are most relevant when comparing vendors. Additionally, models evolve rapidly, which may lead to degradation in performance over time. Version tracking and deliberate postdeployment evaluation are essential to evaluate the phenomenon of model drift rather than relying solely on initial preimplementation testing.

In the absence of standardized evaluation processes, health care provider organizations must rely on vendor-reported testing or conduct their own assessments to understand how a model performs. Vendors are typically best positioned to describe these technical characteristics, including model identity, general training approach, and known performance attributes. Such information can provide a high-level understanding of how well a model handles clinical terminology, accommodates diverse patient populations, or manages ambiguous or noisy inputs. However, transparency varies widely across developers, and the training data most relevant to evaluating generalizability, such as the demographic composition of clinical transcripts used during model development, are often only partially disclosed. Similarly, reporting of ASR-specific performance is limited and lacks standardization, despite its critical role in accurately capturing spoken language across diverse accents, dialects, and clinical environments.

Given this lack of standardization and variability in relevance, health care provider organizations may benefit from supplementing vendor information with internal testing when resources allow. Approaches include structured batch testing with standardized vignettes, automated similarity or factuality metrics, and targeted expert review of outputs to identify context-dependent errors. Public benchmarks such as HealthBench offer a baseline for comparison but do not eliminate the need for local verification [12-14]. Because models evolve rapidly through updates to prompts, parameters, and foundational components, version tracking and reproducible testing protocols are essential for health care provider organizations to maintain an accurate understanding of model behavior. Rapid, iterative assessment, such as simulation testing [15], is therefore required for performance metrics to remain representative of the current product. While such assessments are typically done internally by vendors, they should also be conducted by health care provider organizations independently, and in collaboration with vendors, where feasible. However, we also recognize that this may not be possible for the majority of health care delivery organizations in the United States today, particularly those that are not major academic institutions. Barring established regulatory standards or public release of performance metrics by vendors, most health care delivery organizations will have to either invest in capabilities to conduct internal testing or rely on results shared by academic centers and vendors.

### Level 2: Interface Dimension

The interface dimension addresses how clinicians encounter, interpret, and act on AI-generated content. Even when an underlying model performs well, the design of the interface through which clinicians review, edit, and finalize draft notes plays a central role in shaping real-world performance. This is constrained by several key factors. First, electronic health records (EHRs) are the primary interface for health care delivery, and their design influences cognitive load, situational awareness, error detection, and the overall efficiency of clinical documentation. This reality constrains the clinicians and also limits the potential solutions developed by vendors, as these solutions must integrate with EHRs to fit within existing clinical workflows. There is growing concern that consolidation in the EHR market will negatively influence the ability to address some of the shortcomings of EHRs by stifling much-needed innovation [16]. Health systems need to understand the strengths and limitations of both the EHRs and the ambient AI tools so they can interpret user-facing performance issues, identify usability barriers that may contribute to error or burden, and determine whether a tool is appropriate for their setting. This awareness also informs implementation and training efforts by helping organizations anticipate the cognitive demands clinicians may experience when interacting with a new ambient AI tool.

The first step after understanding the EHR interface and its challenges is assessing the interface of the ambient AI tool and understanding the design characteristics that structure clinicians’ interaction with AI-generated content. These characteristics include the layout and organization of draft notes, the prominence given to key clinical elements, the pathways for editing or accepting suggestions, and the visual cues (such as formatting, color, or iconography) that guide a user’s attention. Although vendors develop these interface features, they do so with substantial constraints. Integration policies and technical architectures of major EHR systems, including Epic, Oracle Health (Cerner), Meditech, and athenahealth, impose specific requirements that shape what can be displayed, how information can be organized, and how clinicians can interact with third-party applications. As a result, interface differences across ambient AI tools may reflect the underlying EHR environment as much as vendor design decisions, and the same product may behave differently across health care provider organizations.

Vendors can generally demonstrate interface features and describe the rationale for certain design choices. However, few provide systematic usability data or human factors evidence documenting how clinicians navigate the interface, detect errors, or manage cognitive load. In many cases, interface decisions are iterative, proprietary, and updated frequently, limiting the extent to which vendor materials alone can inform assessment.

Given this limited transparency and the variability introduced by different EHR environments, health care provider organizations may benefit from conducting local usability evaluations. Methods such as heuristic evaluation (expert review based on established design principles), cognitive walk-throughs (step-by-step task analysis from the user’s perspective), simulation-based testing, and structured observation can reveal whether the interface supports efficient review or introduces unintended safety or workflow concerns. These evaluations help distinguish issues rooted in interface design from those arising from technical model limitations or broader system-level factors and can inform decisions about procurement, training, and implementation.

### Level 3: System-Level Dimension

The system-level dimension encompasses the organizational, social, and environmental factors that shape how an ambient AI technology functions once introduced into an existing clinical environment. Even when a model performs well and the interface supports efficient review, in-situ performance is influenced by how the technology interacts with clinical workflows, staffing patterns, documentation norms, physical environments, and institutional support structures. Ambient AI becomes one component of a broader sociotechnical system, and its effectiveness depends on how well the technology aligns with, adapts to, and influences that system in everyday use. Awareness of these interactions helps health care provider organizations interpret observed performance issues, understand where local workflow or cultural factors may shape outcomes, and determine what forms of training or organizational support are needed for safe and sustained adoption.

Assessing the system-level dimension centers on examining how the technology integrates into ongoing clinical work. This integration includes how clinicians incorporate AI-generated drafts into their documentation routines; how audio capture functions in real clinical rooms; how the tool fits into the pacing and structure of visits; and how responsibilities for reviewing, editing, or correcting AI-generated content are distributed among clinical staff. These factors are not prerequisites to adoption; rather, they are the dynamic conditions that emerge as ambient AI becomes part of the daily workflow. System-level performance reflects these interactions and often explains why the same product performs differently across sites, even when the underlying technology is identical.

Key performance metrics in the system-level dimension include documentation burden reduction, time spent on documentation, coding acuity, patient throughput, and related operational outcomes. While vendors are attentive to these indicators, their capacity to measure them is limited by their lack of access to operational and workflow data within health care provider organizations. As a result, vendor monitoring often relies on indirect indicators such as use rates or engagement metrics. Nevertheless, vendors can support system-level integration by providing onboarding materials, recommended workflows, and insights gathered from prior deployments that may help health care provider organizations anticipate common challenges. Opportunities for health care provider organizations and vendors to collaborate on implementation science, that is, assessing and developing specific interventions to improve the effectiveness of implementation, may be valuable to maximize clinical and operational outcomes.

As system-level performance depends on how the technology interacts with the localized work system, health care provider organizations must conduct their own system-level evaluations to understand real-world performance. This type of assessment often requires a mixed methods approach. Quantitative data may include changes in documentation time, note completeness, coding patterns, message turnaround times, or visit length. Qualitative insights gained through workflow observations, interviews, focus groups, and user experience surveys help reveal adoption patterns, sources of variation, and operational or cultural barriers. In-situ pilot testing in live clinical environments is critical as system-level behaviors cannot be meaningfully inferred from simulations, vendor demonstrations, or usability sessions alone. These evaluations help clarify whether performance issues reflect technical limitations, interface barriers, or site-specific workflow patterns and may be able to guide needed adaptations to support effective use. These assessments can further clarify potential variability in effectiveness across different specialties, visit types, or clinical scenarios to help determine where ambient AI is effective, where additional onboarding may be needed, and where the technology may be poorly suited and should not be deployed. Prior studies of ambient AI provide useful early insights into how ambient AI functions in practice and offer potential methodological approaches for gathering relevant quantitative and qualitative data [17,18]. However, findings from early reports, particularly those from large institutions, may not generalize to other small or medium-sized health care provider organizations that deliver the majority of care across the country.

Financial outcomes are another important aspect of this dimension that should be considered when making deployment decisions. Health systems, especially those in rural settings, often function on thin margins and have real opportunity costs of spending limited resources on this technology. Assessing the costs and benefits, including total cost of ownership, needs to be a key part of core implementation metrics to ensure that financial viability is taken into account before final procurement decisions are made.

### Sequencing and Prioritization Across Dimensions

The sequence of the framework, starting with the technical dimension and ending with the system-level dimension, reflects the increasing resources required to assess each dimension. Technical assessments can be conducted rapidly without dependence on the interface or local workflows; interface assessments require considering human factors in relation to the tool, and system-level assessments typically require the most resource-intensive assessment in live clinical environments. Organizations may therefore benefit from beginning with the least resource-intensive forms of assessment before progressing to more contextualized and operationally demanding assessments. However, this sequencing should not be interpreted as a fixed hierarchy of importance. Severe deficiencies in any dimension may outweigh strengths in the others and should prompt closer scrutiny, targeted mitigation, limitation of deployment to appropriate settings, or nonadoption when the technology does not perform adequately in the intended context.

## Development and Positioning of the Tri-Level Model

The tri-level model was developed as an expert-informed conceptual synthesis rather than through a formal consensus or systematic review process. The model was shaped through iterative discussion among authors with complementary expertise in human factors, clinical implementation, AI product development, and health care operations. Its structure was informed by 3 primary sources: foundational sociotechnical and implementation frameworks, including the Systems Engineering Initiative for Patient Safety (SEIPS) [19] and the Consolidated Framework for Implementation Research (CFIR) [20]; emerging literature on ambient AI tools and AI evaluations; and the authors’ applied experience evaluating, implementing, and studying AI-enabled technologies in clinical settings.

The model is not intended to replace broader and more comprehensive frameworks such as SEIPS or CFIR. Rather, it translates several of their core principles—such as human-centered design, work-system thinking, and attention to local implementation context—into a practical, grounded, domain-specific structure for evaluating ambient AI documentation tools. The resulting framework should therefore be understood as a focused complement to existing models, offering health systems and vendors a shared vocabulary for assessing ambient AI performance.

## Discussion

As ambient AI documentation tools mature and enter routine clinical use, health care provider organizations, researchers, and vendors face the challenge of understanding these technologies within a landscape marked by rapid iteration, variable transparency, and limited standardization. Decisions about adoption, evaluation, and governance are often made without a clear structure for interpreting what constitutes “performance,” which aspects of a tool can be assessed independent of local context, and which aspects must be understood through real-world deployment. System-level outcomes reported by early adopters are difficult to generalize, while technical claims made by vendors are not easily compared across products. The tri-level shared mental model presented in this manuscript—encompassing technical, interface, and system-level dimensions—offers a way to address these challenges by organizing the assessment of ambient AI technologies into meaningful domains. By clarifying these dimensions, the framework supports health care provider organizations to engage in more efficient and informed preimplementation decision-making, provides guidance for ongoing monitoring and governance, and establishes a shared basis for aligning expectations across stakeholders. To illustrate how the framework could be used by a health care provider organization, Textbox 1 presents a hypothetical vignette of a health system deciding between 2 different ambient AI products.

Textbox 1. **Applying the framework in practice: a health system vignette.**

- A regional health care provider organization is deciding between 2 ambient AI documentation vendors for use in primary care and outpatient specialty clinics. Both vendors report high clinician satisfaction, reduced documentation burden, and strong note-generation performance at other health care organizations. However, the available information is not directly comparable: vendor A provides detailed technical documentation but limited usability data, while vendor B provides strong testimonials and workflow case studies but less information about model performance, automatic speech recognition (ASR) accuracy, and update practices. Using the tri-level framework, the organization structures its comparison across technical, interface, and system-level dimensions.
- At the technical level, the organization requests comparable information from both vendors about the underlying ASR and AI models, model versioning, update cadence, prompting or orchestration strategies, and available performance testing. As the organization serves a diverse patient population, it specifically asks each vendor about ASR performance across accents, dialects, multilingual speech, background noise, and multispeaker encounters. It also asks how each vendor measures hallucinations, omissions, factual accuracy, latency, robustness, and performance drift over time. Where vendor-reported information is incomplete or not comparable, the organization conducts limited internal testing using representative encounters or standardized clinical vignettes to assess whether errors appear related to transcription quality, downstream summarization, or unsupported model-generated content.
- At the interface level, the organization compares how clinicians would review, edit, and finalize AI-generated documentation in each product. Human factors or clinical informatics staff conduct brief heuristic evaluations and cognitive walk-throughs of both systems, focusing on note layout; visibility of important clinical information; ease of editing, review burden; and whether the interface helps clinicians detect omissions, hallucinations, or inaccuracies. In simulation-based testing, clinicians review draft notes from both vendors and provide feedback on which interface better supports efficient and accurate review. This comparison may reveal that 2 products with similar technical performance create very different clinician experiences; for example, one may produce accurate notes but present them in a dense format that makes error detection difficult, while another may provide clearer organization, visual cues, or editing pathways that better support clinician oversight.
- At the system level, the organization pilots both tools in a small number of clinical settings with different workflows and documentation norms. Quantitative measures include documentation time, after-hours electronic health record use, note completion time, coding patterns, use rates, and visit length. Qualitative feedback from clinicians and staff is collected to understand how each tool fits into the pacing of visits, whether audio capture works reliably in real exam rooms, and whether clinicians have sufficient time to responsibly review and correct outputs. This evaluation may show that one vendor performs better in routine primary care visits, while another is better suited to a specialty clinic with more structured documentation needs. It may also reveal that one product requires substantially more onboarding or workflow adaptation than the other.
- By applying the framework comparatively, the health care provider organization avoids treating vendor-reported performance as a single global indicator of product quality. Instead, it can distinguish whether differences between products arise from the underlying model, the usability of the interface, or the fit between the tool and local workflows. This structured comparison supports a more informed decision about which vendor to select, where the selected product should be deployed, what additional assurances are needed from the vendor, and what local monitoring should occur after implementation.

A key advantage of this framework is that it differentiates the distinct forces shaping ambient AI performance, which allows evaluators to understand how and when each dimension should be assessed. Documentation issues that appear similar on the surface may arise from very different sources; for example, a technical limitation in how the model handles specialty-specific terminology, an interface design that obscures key information, or a workflow pattern that limits the time clinicians can devote to review. As these influences differ in their dependencies and stability, they require different approaches to evaluation: technical characteristics can often be examined quickly and iteratively, interface characteristics benefit from deliberate user-centered assessment, and system-level outcomes emerge only through observation in real clinical environments. Recognizing these differences enables health care provider organizations and researchers to focus evaluative effort where it is most informative and impactful, rather than relying solely on system-level results that may not apply to their own settings.

Health care provider organizations vary widely in their capacity to evaluate ambient AI technologies, and these differences shape how the framework can be applied in practice. Larger organizations may have the analytic, human factors, and implementation resources needed to conduct substantial technical testing, structured usability evaluations, and system-level pilots. In contrast, small and medium-sized organizations, which provide the majority of health care across the United States, may rely more heavily on vendor-provided materials or external evaluations. As the technical and interface dimensions can be evaluated independently of local workflows, there is potential for shared or external resources such as standardized, comparative assessments to support decision-making across organizations of all sizes. In the absence of such resources, health care provider organizations may conduct their own assessments, particularly for these 2 dimensions, recognizing that they can be meaningfully compared across products. Vendors can contribute valuable information—especially at the technical level, where they can describe model characteristics, training data, and known performance attributes—but have progressively less visibility into interface-related usability challenges and minimal insight into system-level outcomes, which are shaped by local workflows, environments, staffing patterns, and organizational culture. This uneven distribution of evaluative responsibility reinforces the value of a collaborative, layered approach that draws on both vendor insights and local assessment to build a comprehensive understanding of ambient AI performance that begins before selection and continues throughout deployment.

## Risks of Inadequate Performance Assessment

While ambient AI adoption has increased substantially in recent years, important medicolegal and governance risks remain for clinicians and health care delivery organizations due to suboptimal performance of these technologies. All 3 dimensions can result in outputs that increase legal risk for clinicians. There are no established standards for audit trails of clinical note generation today. On the basis of precedent, the clinician is responsible for finalizing content that is entered in the EHR. However, clinicians face multiple challenges that limit their ability to identify erroneous output. They face automation bias, automation complacency, and lack of time and training to take on the role of fact-checker [21-24]. Information gathered through assessment across all 3 dimensions, including the technology’s error patterns, the usability of the review interface, and the system’s fit with local workflows, can help clinicians better anticipate, identify, and correct erroneous output, while clarifying where additional safeguards or support may be needed to reduce automation bias and complacency. Organizations also need to take into account any vendor indemnification clauses in their contracts that relate to how the technology is deployed in the clinical setting. As these confidential documents are specific to organizations and vendors, no specific actions can be recommended in this manuscript beyond generating greater awareness of these issues.

## Alignment With Emerging Standards

This tri-level model provides a practical mechanism for operationalizing key domains of The Joint Commission’s new Responsible Use of AI in Healthcare (RUAIH) certification [25,26], which addresses an organization’s ability to monitor, evaluate, and validate safety performance, effectiveness, and responsible use of AI technologies. This framework offers a structure for determining which metrics should be tracked at each level, how frequently evaluation should occur, and which stakeholders should be responsible for oversight. This includes version tracking, reproducible testing protocols, systematic usability assessment, and evaluation of operational outcomes in live clinical settings. As vendors update prompts, parameters, or foundational models and as clinician behavior evolves over time, health care provider organizations must implement continuous monitoring. This model provides guidance on how health care provider organizations can translate high-level responsible AI expectations, such as those outlined in RUAIH, into concrete evaluation activities.

## Limitations

While we propose this shared mental model as a starting point for health system leaders and industry to use in assessing ambient AI technologies, it has specific limitations. First, we have not included the patient or clinician voice. Second, we also have not included patient safety assessments, ethics, privacy, or data security, which are important factors in selecting and adopting any new technology in health care. These are all areas that need further investigation. Additionally, further research is needed to create and validate the metrics of assessment for each dimension and understand the applicability of this model. Regardless, we think this model can help industry and health care delivery organizations develop a shared understanding of the challenges of adopting solutions in this new product category of ambient AI, which is a new and unique area of technology in health care.

## Conclusions

Ambient AI technologies are integrated into complex sociotechnical systems. Their performance depends on the interaction of underlying model behavior, user-interface design, and local clinical environments. By providing a structured way for health care provider organizations and vendors to conceptualize these interactions and sequence their evaluations, the tri-level model can support more transparent, safe, and effective adoption across organizations of all sizes.

## Abbreviations

ASR: automatic speech recognition
CFIR: Consolidated Framework for Implementation Research
EHR: electronic health record
LLM: large language model
RUAIH: Responsible Use of AI in Healthcare
SEIPS: Systems Engineering Initiative for Patient Safety

## References

[1] Yan S, Knapp W, Leong A (2024). Prompt engineering on leveraging large language models in generating response to InBasket messages. J Am Med Inform Assoc.

[2] Shah SJ, Devon-Sand A, Ma SP (2025). Ambient artificial intelligence scribes: physician burnout and perspectives on usability and documentation burden. J Am Med Inform Assoc.

[3] Afshar M, Baumann MR, Resnik F (2025). A pragmatic randomized controlled trial of ambient artificial intelligence to improve health practitioner well-being. NEJM AI.

[4] Lukac PJ, Turner W, Vangala S (2025). Ambient AI scribes in clinical practice: a randomized trial. NEJM AI.

[5] Tan JY, Rafi IB, Sng GG (2026). Impact of an ambient AI scribe among clinicians and patients: real-world prospective observational time-motion study. JMIR Med Inform.

[6] Bracken A, Reilly C, Feeley A, Sheehan E, Merghani K, Feeley I (2025). Artificial intelligence (AI) - powered documentation systems in healthcare: a systematic review. J Med Syst.

[7] Kim E, Liu VX, Singh K (2025). AI scribes are not productivity tools (yet). NEJM AI.

[8] Sasseville M, Yousefi F, Ouellet S (2025). The impact of AI scribes on streamlining clinical documentation: a systematic review. Healthcare (Basel).

[9] Coiera E, Fraile-Navarro D (2026). AI scribes: are we measuring what matters?. JMIR Med Inform.

[10] Anderson TN, Mohan V, Dorr DA, Ratwani RM, Biro JM, Gold JA (2025). Evaluating the quality and safety of ambient digital scribe platforms using simulated ambulatory encounters. Mayo Clin Proc Digit Health.

[11] Biro J, Handley JL, Cobb NK (2025). Accuracy and safety of AI-enabled scribe technology: instrument validation study. J Med Internet Res.

[12] (2025). Introducing HealthBench. OpenAI.

[13] Arora RK, Wei J, Hicks RS (2025). HealthBench: evaluating large language models towards improved human health. arXiv.

[14] Liu J, Liu S (2025). HealthBench: advancing AI evaluation in healthcare, but not yet clinically ready. Digit Health.

[15] Biro JM, Handley JL, Mickler J (2025). The value of simulation testing for the evaluation of ambient digital scribes: a case report. J Am Med Inform Assoc.

[16] Abulibdeh R, Crowson MG, Douglas MJ, Ramos M, Saillant NN, Celi LA (2026). A problem of Epic proportion. PLOS Digit Health.

[17] Tierney AA, Gayre G, Hoberman B (2025). Ambient artificial intelligence scribes: learnings after 1 year and over 2.5 million uses. NEJM Catal Innov Care Deliv.

[18] Olson KD, Meeker D, Troup M (2025). Use of ambient AI scribes to reduce administrative burden and professional burnout. JAMA Netw Open.

[19] Holden RJ, Carayon P, Gurses AP (2013). SEIPS 2.0: a human factors framework for studying and improving the work of healthcare professionals and patients. Ergonomics.

[20] Damschroder LJ, Reardon CM, Widerquist MA, Lowery J (2022). The updated Consolidated Framework for Implementation Research based on user feedback. Implement Sci.

[21] Skitka LJ, Mosier KL, Burdick M (1999). Does automation bias decision-making?. Int J Hum Comput Stud.

[22] Goddard K, Roudsari A, Wyatt JC (2012). Automation bias: a systematic review of frequency, effect mediators, and mitigators. J Am Med Inform Assoc.

[23] Parasuraman R, Molloy R, Singh IL (1993). Performance consequences of automation-induced “complacency”. Int J Aviat Psychol.

[24] Dzindolet MT, Peterson SA, Pomranky RA, Pierce LG, Beck HP (2003). The role of trust in automation reliance. Int J Hum Comput Stud.

[25] (2026). Joint Commission releases first of its kind exclusively designed for healthcare organizations, voluntary Responsible Use of AI in Healthcare certification. Joint Commission International.

[26] Responsible use of AI in healthcare. Joint Commission.

